# Progressive Interstitial Lung Disease Following Hematopoietic Stem Cell Transplantation for Combined Immunodeficiency: A Novel Homozygous SLC7A7 Variant Suggestive of Lysinuric Protein Intolerance

**DOI:** 10.7759/cureus.117465

**Published:** 2026-10-05

**Authors:** Sara Babour, Hassani Amale, Radi Abdelilah, Rachid Abilkassem

**Affiliations:** 1 Pediatric Rheumatology, Mohammed V Military Training Hospital, Rabat, MAR; 2 Pediatric Medicine, Mohammed V Military Training Hospital, Rabat, MAR; 3 Pediatric Critical Care, Mohammed V Military Training Hospital, Rabat, MAR

**Keywords:** alveolar proteinosis, combined immunodeficiency, hematopoietic stem cell transplantation, interstitial lung disease, slc7a7, variant of uncertain significance, whole exome sequencing, ysinuric protein intolerance

## Abstract

Lysinuric protein intolerance (LPI) is a rare autosomal recessive metabolic disorder caused by biallelic pathogenic variants in SLC7A7, encoding the y+LAT1 transporter responsible for dibasic amino acid absorption in the intestine and reabsorption in the kidney. LPI can cause severe pulmonary disease, including alveolar proteinosis and fibrosing interstitial lung disease (ILD), which may be indistinguishable from post-transplant pulmonary complications.

We report a case of an eight-year-old boy with a history of T-B-NK+ combined immunodeficiency (CID) successfully treated with allogeneic hematopoietic stem cell transplantation (HSCT), who subsequently developed progressive ILD. Whole exome sequencing (WES) identified a novel homozygous missense variant in SLC7A7 (c.265T>C; p.Cys89Arg), classified as a variant of uncertain significance (VUS) with high in silico pathogenicity scores (REVEL 0.94) and not previously reported in population databases. The variant was absent from gnomAD v4.1.0.

This case underscores the diagnostic challenge of distinguishing LPI-associated pulmonary disease from post-transplant ILD, illustrates the utility of WES in the evaluation of unexplained post-transplant complications, and highlights the importance of plasma and urine amino acid chromatography in the biochemical confirmation of this diagnosis. To our knowledge, this is the first reported case of a homozygous SLC7A7 VUS co-occurring with a prior diagnosis of CID and HSCT, raising the possibility that the pulmonary phenotype may be attributable to LPI rather than to transplant-related causes alone.

## Introduction

Lysinuric protein intolerance (LPI; OMIM: 222700) is a rare autosomal recessive disorder of dibasic amino acid transport caused by biallelic loss-of-function variants in SLC7A7, encoding the light chain subunit y+LAT1 of the heterodimeric basolateral amino acid transporter [1,2]. With an estimated prevalence of 1 in 60,000 in Finland and lower elsewhere, LPI is globally underdiagnosed [2,3]. This defect impairs intestinal absorption and renal reabsorption of lysine, arginine, and ornithine, resulting in severely reduced plasma levels and paradoxically elevated urinary excretion of these dibasic amino acids - a biochemical profile highly characteristic of LPI and detectable by urine amino acid chromatography [1]. The consequent deficiency of ornithine and arginine impairs urea cycle function, producing hyperammonemia, while increased carbamyl phosphate flux into the pyrimidine pathway elevates urinary orotic acid [2,4].

The clinical phenotype of LPI is broad and multi-systemic, encompassing growth retardation, hepatosplenomegaly, hematological abnormalities, and pulmonary disease [2,4]. Pulmonary involvement in LPI ranges from fibrosing interstitial lung disease (ILD) to alveolar proteinosis and constitutes the most life-threatening complication of the disorder, occurring in approximately 15%-20% of reported cases [5,3]. LPI is not a recognized classical cause of T-B-NK+ combined immunodeficiency (CID), and in some patients, the two disorders may represent independent genetic conditions. Several cases of immune dysregulation, including hemophagocytic lymphohistiocytosis-like conditions, have been reported in LPI patients [6,3].

Following hematopoietic stem cell transplantation (HSCT), children with prior CID are at risk of multiple pulmonary complications, including bronchiolitis obliterans, organizing pneumonia, chronic pulmonary graft-versus-host disease (GvHD), post-infectious fibrosis, and drug-related toxicity [7]. These diagnoses may coexist with an underlying metabolic pulmonary disease and may be mistaken for each other, particularly when the metabolic disorder has not yet been recognized.

The coexistence of LPI, CID, HSCT, and progressive ILD appears to be exceptionally uncommon. To the best of our knowledge, based on a systematic search of PubMed, ClinVar, gnomAD, and LOVD using the terms 'lysinuric protein intolerance,' 'SLC7A7,' 'combined immunodeficiency,' and 'interstitial lung disease,' no similar case has been previously identified; however, we cannot exclude unreported cases.

We report this case to describe a possible association between a novel homozygous SLC7A7 variant and progressive ILD in a child with a history of HSCT for CID and to highlight the diagnostic value of metabolic investigation in children with unexplained progressive pulmonary disease, growth failure, and consanguinity.

## Case presentation

Patient background and initial presentation

The patient is an eight-year-old male, the second child of first-degree consanguineous parents from El Jadida, Morocco. His birth weight was 3,200 g following an uncomplicated vaginal delivery at term. The family history was otherwise unremarkable, with one healthy elder sibling who later served as the bone marrow donor.

At three months of age, the patient developed acute respiratory distress with prolonged cyanosis without fever or gastrointestinal symptoms. He was hospitalized twice at a regional hospital before being transferred to Casablanca University Hospital. Cytomegalovirus (CMV) interstitial pneumonia was diagnosed on the basis of a positive quantitative polymerase chain reaction (PCR) on peripheral blood/plasma, demonstrating significant CMV viremia. The patient was treated with intravenous ganciclovir, with clinical improvement. We acknowledge that this early CMV pneumonitis may have contributed to subsequent pulmonary remodeling.

At eight months of age, anthropometric measurements revealed severe growth retardation (height, 60 cm, -3 standard deviations (SDs), according to WHO Child Growth Standards; weight, 5 kg, -3 SD).

Diagnosis of CID and HSCT

Immunological workup at eight months of age demonstrated T(functional-) B- NK+ CID (Table 1). Although total CD3+ T cell counts were within the quantitative normal range, qualitative and functional evaluation revealed collapse of naïve T cells (CD4+CD45RA+ <10%; reference, >65%-70%), oligoclonal memory T cell expansion (>90% memory), and a major proliferative defect on phytohemagglutinin (PHA) stimulation (<15% of control; reference, >70%). B cell lymphopenia was severe (CD19+ 120/µL; reference, 600-3,000/µL). NK cells were elevated (2,550/µL). Immunoglobulin levels showed marked hypogammaglobulinemia (IgG 1.2 g/L, IgA <0.05 g/L, IgM 0.18 g/L), and vaccine responses to tetanus and Haemophilus influenzae type b (Hib) antigens were absent. HIV serology was negative.

**Table 1:** Lymphocyte Immunophenotyping at Diagnosis (Age 8 Months) The diagnosis of T(functional-) B- NK+ combined immunodeficiency is supported by the functional and qualitative immunophenotyping data, not cell counts alone. Total CD3+ T cell counts were within the quantitative normal range; however, naïve T cell collapse, oligoclonal memory expansion, a major proliferative defect on PHA stimulation, severe B lymphopenia, absent vaccine responses, and marked hypogammaglobulinemia collectively confirm combined immunodeficiency. NK: natural killer cells; PHA: phytohemagglutinin; Hib: Haemophilus influenzae type b; IVIg: intravenous immunoglobulin

Parameter	Patient Value	Reference Range (3-12 months)	Interpretation
CD3+ T cells	1,649/µL (38%)	2,300-7,000/µL	Moderate quantitative T lymphopenia
CD4+ T cells	1,034/µL (24%)	1,500-5,000/µL	Below age median
CD8+ T cells	565/µL (13%)	600-2,200/µL	Lower limit of normal
CD19+ B cells	120/µL (2.8%)	600-3,000/µL	Severe B lymphopenia
NK cells (CD16+/CD56+)	2,550/µL (59%)	200-1,200/µL	Elevated - NK+
Naïve T cells (CD4+CD45RA+)	<10%	>65-70%	Severe naïve T cell deficiency
Memory T cells (CD4+CD45RO+)	>90%	<30%	Oligoclonal memory expansion
PHA proliferation	<15% of control	>70% of control	Major proliferative defect
IgG (g/L)	1.2	2.0-9.0	Marked hypogammaglobulinemia
IgA (g/L)	<0.05	0.15-0.70	Undetectable
IgM (g/L)	0.18	0.40-1.40	Reduced
Vaccine responses (tetanus/Hib)	Negative	Seroconversion expected	Specific humoral defect
HIV serology	Negative	-	No HIV infection

The patient was initiated on intravenous immunoglobulin replacement and co-trimoxazole prophylaxis. At 14 months of age, he underwent allogeneic bone marrow transplantation from his human leukocyte antigen (HLA)-identical sister (10/10 match; graft, 7.14 × 10⁸ nucleated cells/kg), with cyclosporine for GvHD prophylaxis. The post-transplant course was complicated by tuberculosis (treated with rifampicin and isoniazid) and Epstein-Barr virus (EBV) reactivation (PCR, 9,496 eq-gen/mL), successfully treated with four cycles of rituximab. Immune reconstitution was confirmed by normalization of lymphocyte subpopulations approximately five years post-transplant, and the patient remained infection-free thereafter.

Progressive ILD

Approximately one year post-transplant, thoracic computed tomography (CT) revealed a bilateral diffuse interstitial syndrome with ground-glass opacities, consolidations, and bilateral air trapping, compatible with parenchymal lung disease. This pattern is nonspecific and was compatible with multiple diagnoses, including post-CMV interstitial fibrosis, bronchiolitis obliterans, organizing pneumonia, and chronic pulmonary GvHD. Bronchoscopy was performed and showed no findings consistent with GvHD. CMV PCR remained negative post-transplant under ganciclovir prophylaxis. We acknowledge, however, that bronchiolitis obliterans, organizing pneumonia, and post-CMV fibrosis cannot be formally excluded without histological sampling.

The patient subsequently developed progressive respiratory insufficiency with digital clubbing. At his most recent follow-up before the biochemical evaluation (age approximately eight years and four months; weight, 16 kg (-3 SD, WHO); height, 117 cm (-2 SD, WHO)), oxygen saturation (SpO2) was 91% on room air. Arterial blood gas analysis showed PaO2 62 mmHg and PaCO2 34.7 mmHg. Spirometry demonstrated a restrictive pattern (FVC 77%, FEV1 72% predicted). IGF-1 was severely low at 25 ng/mL (reference, 88-360 ng/mL), a nonspecific finding consistent with chronic illness, growth failure, or nutritional deficiency. Azithromycin at an immunomodulatory dose and supplemental oxygen were initiated in October 2025.

Oxygen saturation improved from 91% to 98% over the subsequent three months. This improvement occurred during azithromycin and oxygen therapy, prior to biochemical confirmation of LPI in January-February 2026. LPI-specific metabolic treatment (protein restriction and citrulline supplementation) was initiated only after biochemical confirmation. The pulmonary improvement therefore cannot be attributed to LPI-specific treatment.

Whole exome sequencing (WES)

Given the unexplained persistence of progressive ILD after documented immune reconstitution, WES was performed on a peripheral blood sample (Hub for Precision Medicine, Casablanca, Morocco). WES identified a novel homozygous missense variant in SLC7A7 (NM_003982.4:c.265T>C; p.Cys89Arg) at chromosome 14q11.2. The variant is absent from gnomAD v4.1.0 (population frequency not reported) and was not identified in a search of ClinVar, HGMD, and LOVD at the time of analysis. Variant interpretation followed ACMG/AMP guidelines. The REVEL in silico score was 0.94 (threshold, ≥0.6; pathogenic range) [8]. The 3Cnet score was 0.54, which falls below the stated pathogenicity threshold of 0.75 and is therefore not cited as supporting evidence for pathogenicity [9]. The associated disease in the database is LPI (OMIM: 222700). Detailed WES variant information is presented in Table 2.

**Table 2:** Whole Exome Sequencing - SLC7A7 Variant Details ACMG: American College of Medical Genetics and Genomics; AMP: Association for Molecular Pathology; VUS: variant of uncertain significance; LPI: lysinuric protein intolerance; REVEL: Rare Exome Variant Ensemble Learner; 3Cnet: constraint-based computational classifier

Parameter	Detail
Gene	SLC7A7
Transcript	NM_003982.4
cDNA variant	c.265T>C
Protein change	p.Cys89Arg
Genomic position (GRCh38)	chr14:22813134 A>G
Zygosity	Homozygous
gnomAD v4.1.0 frequency	Absent (not reported)
REVEL score [10]	0.94 (threshold ≥0.6; pathogenic range)
3Cnet score [11]	0.54 (below stated threshold of 0.75; not supportive of pathogenicity)
ACMG/AMP classification	Variant of uncertain significance (VUS)
ACMG criteria applied	PM2 (absent from population databases, moderate); PP3 (computational tools support pathogenicity - 3 of 4 excluding 3Cnet, supporting); PP4 (phenotype consistent with LPI, supporting). PS3 NOT applied: amino acid chromatography supports LPI phenotype but does not constitute a variant-specific validated functional assay.
Associated disease (OMIM)	Lysinuric protein intolerance (OMIM: 222700)
Parental segregation	Not performed (significant limitation)

ACMG/AMP variant classification at initial assessment: PM2 (absent from population databases, moderate evidence); PP3 (three of four computational tools predict pathogenicity, excluding 3Cnet, which is below threshold, supporting evidence); PP4 (phenotype, once biochemically supported, consistent with LPI as the established gene-disease association, supporting evidence). PS3 is not applied, as amino acid chromatography demonstrating the LPI biochemical phenotype does not constitute a validated functional assay demonstrating the specific effect of the p.Cys89Arg variant. Overall classification: variant of uncertain significance (VUS), pending parental segregation and functional studies.

Biochemical confirmation

Following the genetic result, targeted biochemical investigations were performed. Amino acid chromatography confirmed a profile highly characteristic of LPI: plasma concentrations of lysine (15.60 µmol/L; reference, 80.00-200.00), arginine (3.10 µmol/L; reference, 4.45-56.51), and ornithine (25.40 µmol/L; reference, 53.15-142.33) were markedly reduced, while urinary excretion of the same amino acids was paradoxically and markedly elevated, reflecting defective SLC7A7-mediated basolateral transport with renal tubular wasting. The complete plasma and urine amino acid chromatography results are presented in Table 3. Serum ammonia was elevated at 85 µmol/L (reference, 11-35 µmol/L), reflecting urea cycle dysfunction secondary to deficient ornithine and arginine availability. Urinary orotic acid was elevated at 5.2 mmol/mol creatinine (reference, <4 mmol/mol creatinine), consistent with carbamyl phosphate overflow into the pyrimidine synthesis pathway [2,4]. Additional biochemical markers, including C-reactive protein (CRP) (85 mg/L), ferritin (203 ng/mL), and lactate dehydrogenase (LDH) (600 IU/L), were elevated. These values represent nonspecific markers of systemic inflammation and tissue injury and are presented in Table 4.

**Table 3:** Amino Acid Chromatography Results (Plasma and Urine) at Biochemical Evaluation The table presents plasma and urine amino acid chromatography results at the time of biochemical confirmation. Plasma concentrations of all three dibasic amino acids are markedly decreased, while urinary excretion of the same amino acids is markedly elevated, constituting the pathognomonic biochemical signature of lysinuric protein intolerance (LPI) caused by defective SLC7A7-mediated basolateral transport. The reference range for urine amino acid values matches the plasma reference range as reported by the laboratory (LRAM, Rabat). ▼: below reference range; ▲: above reference range.

Amino Acid	Plasma (μmol/L)	Plasma Reference Range (μmol/L)	Urine (μmol/L)
Lysine	15.60 ▼	80.00-200.00	285.50 ▲
Arginine	3.10 ▼	4.45-56.51	125.80 ▲
Ornithine	25.40 ▼	53.15-142.33	185.00 ▲

**Table 4:** Key Laboratory Parameters Across the Disease Course This table presents selected laboratory results at three time points: initial diagnosis (2017), post-transplant follow-up (2021), and biochemical confirmation of LPI (2026). Reference ranges are provided for key parameters. Reference ranges: hemoglobin 11.5-15.5 g/dL; white blood cell count 4,500-13,500/mm^3^; lymphocytes 1,500-4,000/mm^3^; platelets 150,000-400,000/mm^3^; CRP <6 mg/L; LDH 120-300 IU/L; ferritin 12-150 ng/mL; IGF-1 88-360 ng/mL (age-appropriate); serum ammonia 11-35 μmol/L. VUS: variant of uncertain significance; N/A: not available at that time point; LDH: lactate dehydrogenase; IGF-1: insulin-like growth factor-1; FVC: forced vital capacity; FEV1: forced expiratory volume in one second; SpO2: oxygen saturation; SD: standard deviation; ↑: above the upper limit of the reference range

Parameter	At Diagnosis (Age 8 Months)	Post-transplant (Years 1-2)	Biochemical Confirmation (Current Admission)	Reference Range
Hemoglobin (g/dL)	13.5	13	Within normal limits	11.5-15.5 g/dL
White blood cell count (/mm^3^)	9,200	8,300	Within normal limits	4,500-13,500/mm³
Lymphocytes (/mm^3^)	5,370	2,490	Within normal limits	1,500-4,000/mm³
Platelets (/mm^3^)	377,000	139,000	Within normal limits	150,000-400,000/mm³
C-reactive protein (mg/L)	7.3	<6	85 mg/L ↑	≤6 mg/L
IgG (g/L)	15.8 (maternal)	8.62	8.62 g/L	7-16 g/L
LDH (IU/L)	747 IU/L (↑)	N/A	600 IU/L ↑	120-300 IU/L
Ferritin (ng/mL)	N/A	268 ng/mL	203 ng/mL ↑	12-150 ng/mL
IGF-1 (ng/mL)	N/A	N/A	25 (severely low; reference: 88-360)	88-360 ng/mL
Serum ammonia (µmol/L)	N/A	N/A	85 μmol/L ↑	11-35 μmol/L
Urinary orotic acid	N/A	N/A	5.2 mmol/mol creatinine ↑	≤4 mmol/mol creatinine
Urinary lysine (µmol/L)	N/A	N/A	285.50 µmol/L (↑)	80-200 μmol/L
Urinary arginine (µmol/L)	N/A	N/A	125.80 µmol/L (↑)	4.45-56.51 μmol/L
Urinary ornithine (µmol/L)	N/A	N/A	185.00 µmol/L (↑)	53.15-142.33 μmol/L
FVC (%)	N/A	N/A	77%	>≥80% predicted
FEV1 (%)	N/A	N/A	79%	>≥80% predicted
SpO2 on room air	N/A	91%	98%	≥95%
SLC7A7 variant	-	-	c.265T>C (p.Cys89Arg); homozygous VUS	N/A

Following biochemical confirmation, dietary protein restriction and oral citrulline supplementation were initiated under specialist metabolic supervision. At the most recent follow-up, SpO2 was 98% on room air (weight, 20 kg; FVC, 77%; FEV1, 79%). The six-minute walk test produced no desaturation. The overall clinical course is summarized in Figure 1.

**Figure 1:**
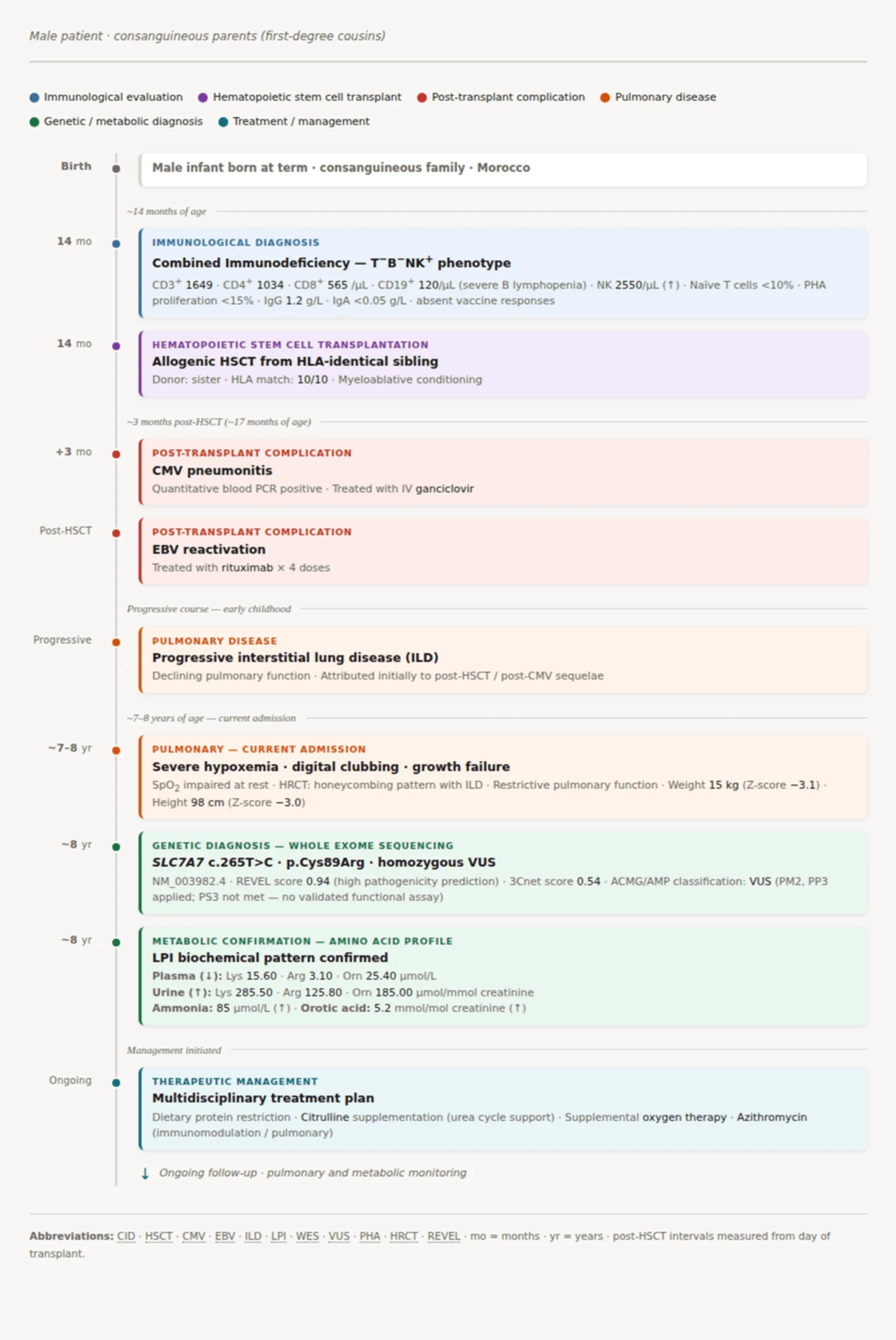
Clinical Chronology and Management Timeline of a Child With SLC7A7-Related Lysinuric Protein Intolerance Presenting as Post-transplant Interstitial Lung Disease Clinical timeline of the patient's course from birth to current admission depicting the sequence of immunological evaluation, hematopoietic stem cell transplantation, post-transplant complications, interstitial lung disease progression, genetic and metabolic diagnosis, and current management. HSCT: hematopoietic stem cell transplantation; HLA: human leukocyte antigen; EBV: Epstein-Barr virus; CMV: cytomegalovirus; VUS: variant of uncertain significance; LPI: lysinuric protein intolerance; PCR: polymerase chain reaction

## Discussion

This case illustrates an important and underappreciated diagnostic challenge: progressive ILD in a child with a prior transplant history is not automatically a post-transplant complication. The seven-year diagnostic delay in this patient underscores the importance of maintaining a broad metabolic differential in children with unexplained pulmonary disease, growth failure, and consanguineous parentage, even when a prior transplant history appears to provide a sufficient explanation.

SLC7A7 and the biochemical profile of LPI

SLC7A7 encodes the y+LAT1 subunit, which forms a heterodimeric cationic amino acid transporter at the basolateral membrane of intestinal enterocytes and renal tubular cells in association with 4F2hc (SLC3A2) [1,2]. Loss of function of y+LAT1 impairs the efflux of dibasic amino acids from epithelial cells into the bloodstream, resulting in defective intestinal absorption and renal wasting [1]. Plasma levels of lysine, arginine, and ornithine are consequently low, while urinary excretion is paradoxically elevated - the biochemical profile highly characteristic of LPI, confirmed in our patient [2]. The arginine and ornithine deficiency impairs urea cycle function, producing hyperammonemia and elevated urinary orotic acid, both present in our patient [4]. WES identified a homozygous variant in the SLC7A7 gene (NM_003982.4): c.265T>C, p.Cys89Arg (Table 5).

**Table 5:** Chronological Summary of Clinical Events, Investigations, and Management The table presents the complete clinical timeline from initial respiratory presentation at three months of age through biochemical confirmation of LPI. ILD: interstitial lung disease; HSCT: hematopoietic stem cell transplantation; WES: whole exome sequencing; LPI: lysinuric protein intolerance; VUS: variant of uncertain significance; CT: computed tomography; EBV: Epstein-Barr virus; GvHD: graft-versus-host disease; SpO2: oxygen saturation; FVC: forced vital capacity; FEV1: forced expiratory volume in one second; MAS: macrophage activation syndrome; LDH: lactate dehydrogenase

Age / Timepoint	Event	Key Findings and Management
Age 3 months	Initial presentation	Acute respiratory distress with cyanosis; cytomegalovirus (CMV) interstitial pneumonia diagnosed. Transferred from regional hospital to Casablanca University Hospital.
Age 8 months	CID diagnosis	T-B-NK+ combined immunodeficiency confirmed. CD3+ 1,649/mm3; CD4+ 1,034/mm3; CD8+ 597/mm3; CD19+ 382/mm3; NK 208/mm3. IgG 15.8 g/L (maternal). Intravenous immunoglobulin + co-trimoxazole prophylaxis initiated.
Age 14 months (Day 1 of transplant)	Allogeneic HSCT	Bone marrow transplant from HLA-identical sibling (10/10 match). Graft: 7.14 x 10(8) nucleated cells/kg. Cyclosporine GvHD prophylaxis.
Post-transplant: years 1-2	Post-transplant complications	Tuberculosis treated with rifampicin and isoniazid. EBV reactivation (PCR: 9,496 eq-gen/mL) treated with four cycles of rituximab with good response. CT thorax: bilateral basal honeycomb interstitial lung disease.
Age ~7 years (stable follow-up)	Stable follow-up	Weight 16 kg; height 107 cm. Clinically stable. Normal lymphocyte subpopulations. No treatment required.
Day 1 of current admission	Worsening ILD	Weight 16 kg (-3 SD); height 117 cm (-2 SD). Digital clubbing; SpO2 91% on room air; PaO2 62 mmHg. FVC 77%; FEV1 72%. IGF-1 25 ng/mL (severely low). Azithromycin (immunomodulatory dose) and home oxygen initiated.
Day 1 of WES and biochemical evaluation	WES performed	Whole exome sequencing: SLC7A7 c.265T>C (p.Cys89Arg), homozygous variant of uncertain significance (VUS). REVEL score: 0.94. Absent from gnomAD v4.1.0. Associated disease: lysinuric protein intolerance (OMIM: 222700).
Pulmonary follow-up (most recent)	Pulmonary follow-up	SpO2 98% on room air. Weight 20 kg. FVC 77%; FEV1 79%. Six-minute walk test: no desaturation.
Day 1 of biochemical confirmation	Biochemical confirmation of LPI	Urine amino acid chromatography: markedly elevated lysine, arginine, and ornithine. Serum ammonia: elevated. Urinary orotic acid: markedly elevated. Ferritin and LDH: markedly elevated, consistent with macrophage activation syndrome (MAS). Biochemical profile pathognomonic of lysinuric protein intolerance.

The biochemical findings in this patient - low plasma dibasic amino acids, elevated urinary dibasic amino acids, elevated ammonia, and elevated urinary orotic acid - are highly characteristic of LPI as a disease phenotype. However, it is important to distinguish this from direct functional evidence for the pathogenicity of the specific p.Cys89Arg variant. Routine amino acid chromatography does not constitute a validated functional assay demonstrating the effect of this particular variant. The variant therefore remains a VUS pending parental segregation analysis and formal functional studies. Plasma dibasic amino acid concentrations were markedly reduced, and urinary excretion was elevated, confirming the LPI biochemical pattern (Table 6).

**Table 6:** Serological Findings and Interpretation The table presents serological and immunological findings across the disease course with their clinical interpretation. CMV: cytomegalovirus; EBV: Epstein-Barr virus; ANA: antinuclear antibodies; anti-dsDNA: anti-double-stranded DNA

Test	Result	Interpretation
HIV serology	Negative	No HIV infection
CMV pneumonia (age 3 months)	Positive (interstitial pneumonia)	Active CMV pulmonary infection; treated with IV ganciclovir
EBV PCR (post-transplant)	9,496 eq-gen/mL (elevated)	EBV reactivation; treated with four cycles of rituximab
Antinuclear antibodies (ANA)	Transiently positive (1/160)	Non-specific; not diagnostic of autoimmune disease
Anti-double-stranded DNA (anti-dsDNA)	Negative	Systemic lupus erythematosus excluded
Anti-transglutaminase IgA	Negative	Celiac disease excluded
Thyroid function tests	Normal	Thyroid disease excluded
Growth hormone stimulation test	Normal	Primary growth hormone deficiency excluded

We note an important methodological limitation: the LRAM laboratory report used the same reference intervals for both plasma and urine amino acid concentrations, which is methodologically inappropriate. Urinary amino acid concentrations should ideally be normalized to urinary creatinine and compared against age-specific urinary reference intervals. This limitation is acknowledged and does not invalidate the directional findings - the combination of markedly low plasma and markedly elevated urinary dibasic amino acids - which remains highly characteristic of LPI regardless of the specific reference intervals applied.

Variant interpretation

The p.Cys89Arg variant (NM_003982.4:c.265T>C) is apparently novel based on a search of ClinVar, gnomAD v4.1.0, HGMD, LOVD, and the published literature at the time of analysis. This appears to be the first reported occurrence of this variant in a patient with a biochemical phenotype highly characteristic of LPI, if the literature search is confirmed to be comprehensive. The applicable ACMG/AMP criteria are PM2 (absent from population databases), PP3 (computational tools supporting pathogenicity, excluding 3Cnet, which is below threshold), and PP4 (phenotype consistent with LPI once biochemically confirmed). PS3 is not applicable, as the biochemical phenotype does not constitute a variant-specific functional assay. The variant therefore remains classified as a VUS. Formal reclassification would require (i) parental segregation analysis confirming that both parents are heterozygous carriers; (ii) a validated cellular functional assay demonstrating the effect of p.Cys89Arg on y+LAT1 transport activity; and (iii) ideally, identification of this variant in additional affected individuals.

Pulmonary disease in LPI: differential diagnosis and uncertainty

Pulmonary involvement is among the most life-threatening manifestations of LPI, ranging from fibrosing ILD to alveolar proteinosis [3,5]. The CT pattern observed in our patient - bilateral ground-glass opacities, consolidations, and air trapping - is nonspecific and compatible with multiple diagnoses. We acknowledge that this pattern does not constitute evidence for pulmonary alveolar proteinosis specifically; the latter classically presents with bilateral ground-glass opacities and a crazy-paving pattern on CT and requires confirmation by bronchoalveolar lavage demonstrating milky fluid rich in periodic acid-Schiff (PAS)-positive material or by granulocyte-macrophage colony-stimulating factor (GM-CSF) autoantibody testing. Neither bronchoalveolar lavage nor histological sampling was performed in our patient, and the diagnosis of pulmonary alveolar proteinosis is not established.

The differential diagnosis of the observed pulmonary phenotype includes (i) LPI-associated fibrosing ILD or alveolar proteinosis; (ii) post-CMV interstitial fibrosis secondary to the initial pneumonitis at age three months; (iii) bronchiolitis obliterans; (iv) organizing pneumonia; (v) chronic pulmonary GvHD; and (vi) conditioning-related toxicity. Bronchoscopy was performed and was negative for GvHD findings; however, bronchiolitis obliterans and organizing pneumonia cannot be formally excluded without histological sampling. LPI and post-transplant pulmonary disease are not mutually exclusive and may coexist. We present LPI as a strongly suspected contributing cause, not as the definitively proven sole etiology.

Relationship between LPI and CID

The relationship between LPI and the CID in this patient is uncertain. LPI is not a recognized classical cause of T-B-NK+ CID. An independent genetic cause of the original immunodeficiency cannot currently be excluded, and the two conditions may represent independent genetic disorders. The hypothesis that arginine depletion secondary to SLC7A7 deficiency could aggravate immune function is biologically plausible - arginine is an essential substrate for lymphocyte proliferation and nitric oxide synthesis - but this remains unproven in this patient. Several previously published LPI cases have described partial immune dysfunction and hemophagocytic lymphohistiocytosis-like conditions [3,6], but a causal relationship between SLC7A7 deficiency and the T-B-NK+ phenotype in our patient cannot be established based on the available evidence.

Nonspecific inflammatory markers

At the time of biochemical confirmation, CRP was 85 mg/L, ferritin 203 ng/mL, and LDH 600 IU/L. These values represent nonspecific markers of systemic inflammation and tissue injury, consistent with multisystem disease. They do not fulfill the HLH-2004 or MAS diagnostic criteria, which require additional findings such as fever, splenomegaly, cytopenias, hypertriglyceridemia, hypofibrinogenemia, markedly elevated ferritin (typically >500 ng/mL), elevated soluble IL-2 receptor, impaired NK cell activity, or hemophagocytosis on marrow examination. A diagnosis of macrophage activation syndrome is not supported by the available data.

Management considerations

The standard management of LPI includes dietary protein restriction combined with oral citrulline supplementation, both of which were initiated in our patient following biochemical confirmation [3]. Protein restriction must be carefully balanced against the risk of exacerbating malnutrition and growth failure in a child with severe pre-existing growth retardation; specialist metabolic supervision is essential. Oral lysine supplementation may also be considered. For LPI-associated alveolar proteinosis, whole-lung lavage and recombinant GM-CSF have been reported in isolated cases, but these interventions are not standard management and are supported only by limited case reports. Long-term surveillance should include monitoring of plasma amino acids, urinary orotic acid, serum ammonia, ferritin, LDH, renal function, and pulmonary function, with clinical parameters adjusted according to specialist metabolic guidance [3].

Limitations

This report has several important limitations that must be acknowledged.

First, parental genetic testing to confirm biallelic inheritance through segregation analysis was not performed. In the context of first-degree consanguinity, parental confirmation that both parents are heterozygous carriers of the SLC7A7 p.Cys89Arg variant would substantially strengthen the genetic interpretation and is the most important outstanding evidence needed for variant reclassification.

Second, functional characterization of the p.Cys89Arg variant in a validated cellular expression system has not been performed. Such studies would provide direct evidence of the variant's effect on y+LAT1 transport activity and would be required before the variant can be confidently reclassified from VUS.

Third, the urinary amino acid reference intervals used in this report are laboratory-specific (LRAM, Rabat) and were identical to the plasma reference intervals - a methodological limitation. Urinary amino acid concentrations should ideally be normalized to urinary creatinine and compared against validated age-specific urinary reference intervals. This does not invalidate the directional findings, which remain highly characteristic of LPI.

Fourth, the pulmonary diagnosis remains uncertain. Bronchoalveolar lavage, PAS staining, GM-CSF autoantibody testing, and lung biopsy were not performed. The diagnosis of pulmonary alveolar proteinosis is therefore not established. Post-transplant causes, including bronchiolitis obliterans, organizing pneumonia, and post-CMV fibrosis, cannot be formally excluded without histological sampling.

Fifth, the genetic basis of the original CID has not been definitively established. An independent primary immunodeficiency with a separate genetic etiology cannot be excluded. Genome reanalysis with an immunological focus has been recommended.

Sixth, follow-up after initiation of LPI-specific treatment (protein restriction and citrulline supplementation) is limited, and objective improvement in pulmonary, nutritional, and metabolic outcomes attributable specifically to LPI-directed therapy has not yet been documented.

Seventh, CT images with formal radiologist-reviewed annotation were not available for inclusion in this manuscript version. This represents an acknowledged limitation; submission of CT images is planned once high-quality digital images are retrieved from the institutional archive.

## Conclusions

LPI should be considered in children with otherwise unexplained progressive lung disease, growth failure, consanguinity, and compatible metabolic abnormalities - including those with a history of HSCT. In this patient, a homozygous SLC7A7 VUS significance, apparently novel and not previously identified in the databases searched, combined with a biochemical profile highly characteristic of LPI, raised strong suspicion of LPI as a possible contributor to the pulmonary phenotype. Nevertheless, parental segregation analysis, validation of the biochemical results using age-appropriate urinary reference intervals with creatinine normalization, and further functional or molecular evidence are required before the variant can be confidently reclassified and causality established. This case highlights the value of integrating genomic findings with carefully validated biochemical investigation in children with unexplained multisystem disease, while emphasizing the importance of interpreting variants of uncertain significance cautiously in the absence of confirmatory evidence.
